# A high-speed single sideband generator using a magnetic tunnel junction spin torque nano-oscillator

**DOI:** 10.1038/s41598-017-13551-5

**Published:** 2017-10-18

**Authors:** Raghav Sharma, Naveen Sisodia, Ezio Iacocca, Ahmad A. Awad, Johan Åkerman, P. K. Muduli

**Affiliations:** 1 Department of Physics, Indian Institute of Technology, Hauz Khas, New Delhi, 110016 India; 2 Department of Physics, Division for Theoretical Physics, Chalmers University of Technology, 412 96 Gothenburg, Sweden; 3 Department of Applied Mathematics, University of Colorado, Boulder, Colorado, 80309 USA; 4 Department of Physics, University of Gothenburg, 41296 Gothenburg, Sweden; 5 Materials and Nanophysics, School of Engineering Sciences, KTH-Royal Institute of Technology, Electrum 229, 164 40 Kista, Sweden

## Abstract

An important property of spin-torque nano-oscillators (STNOs) is their ability to produce a frequency modulated (FM) signal, which is very critical for communication applications. We here demonstrate a novel single sideband (SSB) modulation phenomenon using a magnetic tunnel junction (MTJ)-based STNO, which saves transmission bandwidth and in principle should minimize attenuation for wireless communication. Experimentally, lower single sidebands (LSSBs) have been successfully demonstrated over a wide range of modulation frequency, *f*_*m*_ = 150 MHz-1 GHz. The observed LSSBs are determined by the intrinsic properties of the device, which can be modeled well by a nonlinear frequency and amplitude modulation formulation and reproduced in macrospin simulations. Moreover, our macrospin simulation results show that the range of modulation current and modulation frequency for generating SSBs can be controlled by the field-like torque and biasing conditions.

## Introduction

A nanopillar consisting of two magnetic layers, one free and one fixed, separated by a non-magnetic layer can be used as a tunable radio-frequency (RF) generator by using the concept of spin transfer torque^1,2^. These spin torque nano-oscillators (STNOs)^3–6^ offer immense potential for future communication applications due to their broad frequency tuning range^7–9^, nanoscopic footprint^5^, and straightforward integration with semiconductor technology using the same processes as magnetoresistive random access memory^10,11^. Whereas metallic spin-valve-based STNOs suffer from limitations on output power, magnetic tunnel junction (MTJ) STNOs have been shown to enhance this figure-of-merit, achieving up to 10 μW^12^, which meets the requirements of commercial applications. Another important advantage of STNOs is their ability to produce a modulated signal upon application of a low radio frequency (RF) signal^8,13–19^. Different modulation schemes—such as nonlinear frequency modulation (NFM)^16,20^, nonlinear frequency and amplitude modulation (NFAM)^18,21–23^, amplitude shift keying (ASK), on–off keying (OOK) modulation^24–26^, and frequency-shift keying (FSK) modulation^27^—have been demonstrated in STNOs for communication applications. Recently, the ASK modulation scheme was used to demonstrate wireless communication of STNO signals^24–26^ up to a distance of 100 cm. This demonstration provides an initial breakthrough in the field of signal transmission, and especially for digital signal processing using nanosized STNOs.

In many wireless communication applications, the primary baseband signal is naturally generated in the form of analog signal, such as a voice or audio signal, and additional circuitry is required for the analog-to-digital conversion. This increases the complexity of the circuit. STNOs can be directly used as analog frequency modulators^16,18,21^. However, frequency modulation schemes require twice the bandwidth of the original baseband signal and consume high power as both lower and upper sidebands are transmitted. The single sideband (SSB) modulation is a modulation scheme that requires less power to transmit than conventional amplitude modulation (AM) and occupies only half of the bandwidth required for other modulation schemes, like double-sideband suppressed carrier (DSB-SC). SSB utilizes 25% less bandwidth for transmission than the popular vestigial sideband (VSB) modulation scheme. SSB modulation is used for long-distance transmission, as it allows for longer spacing between repeaters. The International Telecommunication Union (ITU) has recommended using broadcasting with single sideband (SSB) modulation. It is thus desired that all forms of communication be based on SSB. However, traditional SSB modulators like the Hartley^28^ and Weaver modulators^29^ require many circuit components, including low-pass filters, phase shifters, and quadrature mixers. This significantly increases both size and complexity of the modulators.

We here propose an entirely new scheme for SSB modulation, in which a nanopillar of MTJ is used to directly generate an SSB modulation at a high carrier frequency. We demonstrate lower single sideband (LSSB) modulation rates up to 1 GHz, taking advantage of the STNO’s nonlinear properties. This advancement in SSB through STNOs opens up a new dimension of applications that are easily approachable, fast, and practical for on-chip technology. We show that the observed SSB can be quantitatively explained using NFAM theory^18,22^. Furthermore, using macrospin simulations, we show that the field-like torque can be used to manipulate the range of modulation current *I*_*m*_ and modulation frequency *f*_*m*_ for observing the SSB.

## Results

### Devices

**Figure 1:**
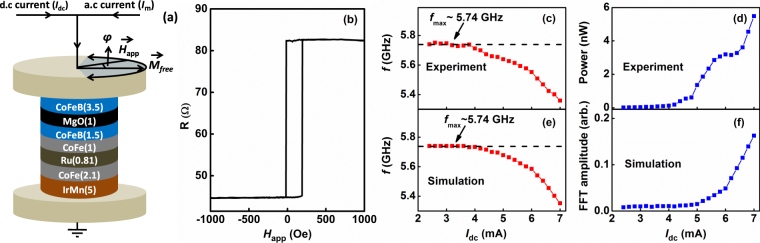
Device structure and free-running properties. (**a**) Magnetic tunnel junction device with a nominal diameter of *D* = 180 nm consists of IrMn (5)/CoFe (2.1)/Ru (0.81)/CoFe (1)/CoFeB (1.5)/MgO (1)/CoFeB (3.5) (thicknesses in nm). (**b**) The Magnetoresistance loop of the MTJ nanopillar showing tunneling magnetoresistance of 84% measured at *φ* = 180°. (**c**) Frequency and (**d**) Power vs. dc current (in the absence of any RF current) measured at *H*_app_ = 450 Oe, *φ* = 190°. (**e**) and (**f**) are the simulation results under the same experimental conditions and at *T* = 300 K.

The device is a MTJ-based STNO nanopillar with a circular cross-section of nominal diameter (*D*) of 180 nm, as shown in Fig. 1(a). The structure of the device is IrMn (5 nm)/CoFe (2.1 nm)/Ru (0.81 nm)/CoFe (1 nm)/CoFeB (1.5 nm)/MgO (1 nm)/CoFeB (3.5 nm). The antiferromagnetic IrMn layer is used to provide an exchange bias on the adjacent CoFe-pinned layer (PL), which couples antiferromagnetically through Ru to the composite CoFe/CoFeB reference layer (RL). The CoFeB layer above the MgO tunnel barrier is the free layer (FL). The RL magnetization is taken to be along the positive *x*-axis. The magnetization of the FL at zero field is antiparallel to the magnetization of the RL, so that the angle between the free and reference layers, defined by *φ* is 180°. The FL magnetization can be coherently rotated with an external magnetic field from 140° to 220°^15^. The resistance–area product in the parallel state is about 1.5 Ω μm^2^. The tunneling magnetoresistance of the device under study is 84% as shown in Fig. 1(b), which shows the magnetoresistance measured along *φ* = 180°. The magnetoresistance show a shift of the hysteresis loop due to interlayer exchange coupling, *H*_*IEC*_ between the FL and RL. The measured *H*_*IEC*_ is about 110 Oe.

### Free running properties

Figure 1(c,d) shows the behavior of the frequency and power versus dc current, measured with an in-plane magnetic field of *H*_app_ = 450 Oe and *φ* = 190°^30^. As expected for this device^30^, the precession corresponds to the magnetization of the FL, for which the frequency red-shifts and power increases very rapidly with dc current. Figure 1(e,f) shows the corresponding simulated behavior of the FL frequency and power versus dc current, which shows excellent agreement with experiment. An important property of this free-running behavior is the presence of a maximum frequency of operation of the STNO, *f*_*max*_ ~ 5.74 GHz for both experiment as well as simulation. As we will show below, this property is essential for observing LSSB.

### Experimental results of LSSB generation

**Figure 2:**
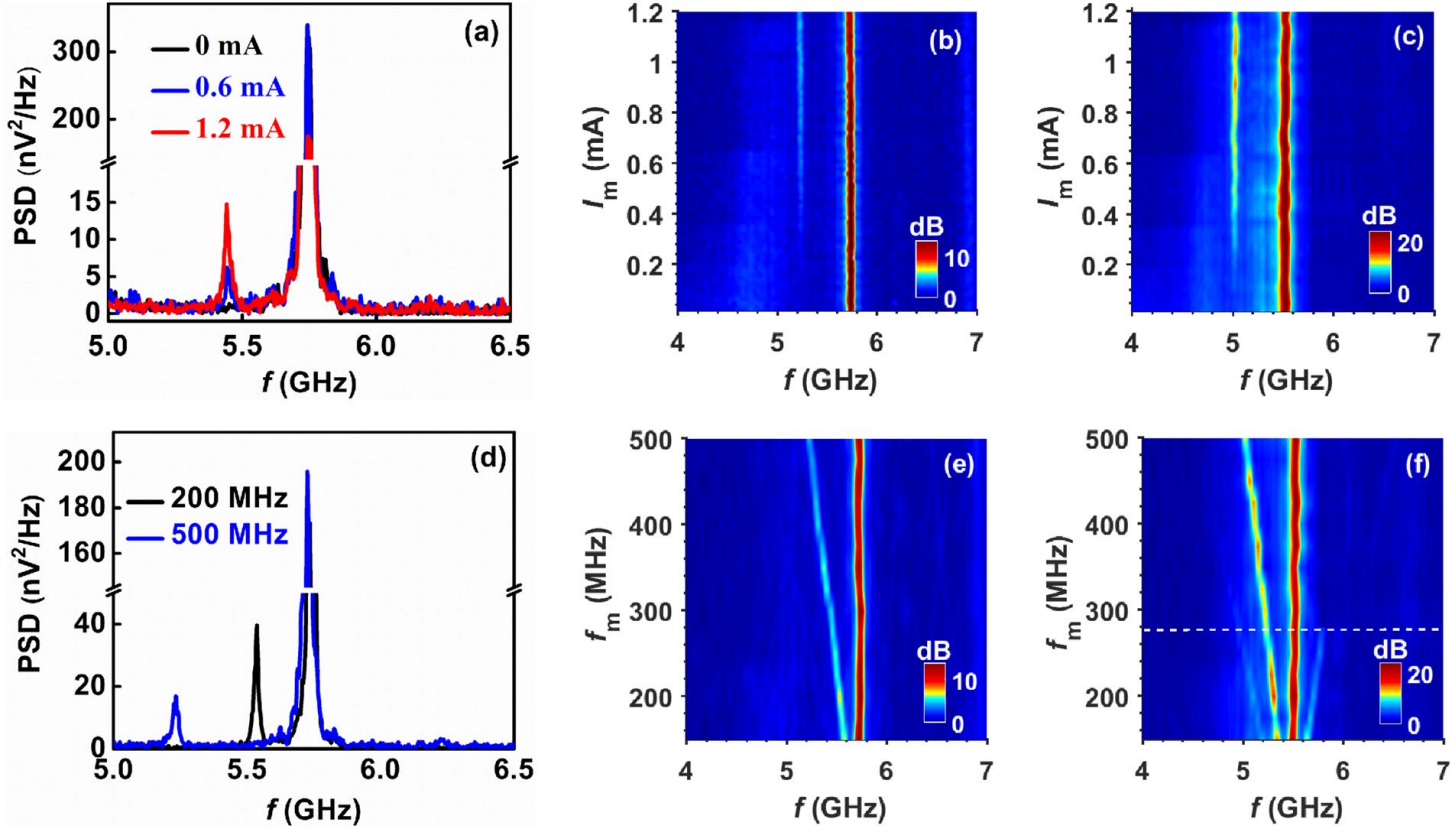
Lower single sideband modulation. (**a**) Sample spectrum showing single sideband modulation at *I*_dc_ = 4.4 mA for different modulation currents. Map of power vs. frequency and modulation current for *f*_*m*_ = 500 MHz at (**b**) *I*_dc_ = 4.4 mA and (**c**) *I*_dc_ = 6.4 mA. (**d**) Sample spectrum showing single sideband modulation at *I*_dc_ = 4.4 mA for different modulation frequencies. Map of power vs. frequency and modulation frequency for *I*_*m*_ = 1.2 mA at (**e**) *I*_dc_ = 4.4 mA and (**f**) *I*_dc_ = 6.4 mA. The white dashed line in (f) separates the NFAM and LSSB regions.

In Fig. 2, we show how lower single sideband (LSSB) modulation is produced when an additional RF signal is superimposed over the dc current (*I*_dc_). The additional RF signal with relatively low frequency is analogous to the information that needs to be sent with the high-frequency carrier generated by the STNO. LSSB generation is shown in Figs 2(a–f) at varying modulation currents and modulation frequencies, respectively. LSSB generation is characterized by the dramatic disappearance of the upper sideband from the spectrum. Figure 2(a) shows the sample spectra at *I*_dc_ = 4.4 mA for different modulation currents (*I*_*m*_), showing only the lower sideband. Figure 2(b,c) shows the frequency spectrum as a function of *I*_*m*_ at *f*_*m*_ = 500 MHz for two examples of dc-biasing currents of *I*_dc_ = 4.4 mA and *I*_dc_ = 6.4 mA. Figure 2(d) shows the spectra at *I*_dc_ = 4.4 mA for different values of *f*_*m*_, whereas Fig. 2(e,f) shows the frequency spectrum for *f*_*m*_ = 150–500 MHz at *I*_*m*_ = 1.2 mA at dc biasing currents of *I*_dc_ = 4.4 mA and *I*_dc_ = 6.4 mA. The threshold current (*I*_th_) for auto-oscillations in this device is about 6 mA^31,32^. Hence *I*_dc_ = 4.4 mA is in the sub-threshold region, whereas *I*_dc_ = 6.4 mA is above the threshold region. The signals obtained at *I*_dc_ = 4.4 mA are attributed to thermally driven ferromagnetic resonance signals^4^. Hence, the power of the carrier and sidebands in the case of *I*_dc_ = 4.4 mA is much less than for *I*_dc_ = 6.4 mA.

For the case of *I*_dc_ = 4.4 mA, LSSB is obtained over the entire range *f*_*m*_ = 150–500 MHz [Fig. 2(e)], whereas, for *I*_dc_ = 6.4 mA, LSSB is obtained for *f*_*m*_ > 275 MHz as shown by the white dashed line in Fig. 2(f). This can be explained on the basis of *f*_*max*_ observed in Fig. 2(c) and (d). For *I*_dc_ = 4.4 mA, the STNO is modulated close to *f*_*max*_ so that the frequency of the upper sideband falls in the region where no mode is allowed. Hence, we get the LSSB modulation for *f*_*m*_ = 150–500 MHz at *I*_dc_ = 4.4 mA. In the case of *I*_dc_= 6.4 mA, the carrier frequency (*f*_0_) is ~275 MHz far from *f*_*max*_, which explains the higher threshold *i*.*e*.,  *f*_*m*_ = 275 MHz for the observation of clear LSSB. Thus the onset frequency for generation of LSSB is directly related to the difference (*f*_*max*_ − *f*_0_) [See supplementary information], which are ~65 MHz and ~272 MHz, for *I*_dc_= 4.4 mA and *I*_dc_= 6.4 mA, respectively. Hence the SSB operating region strongly depends on the value of dc current, and operating above *I*_th_ shifts the onset of SSB to higher *f*_*m*_ for a given *I*_*m*_.

Detailed study shows that SSB can be achieved up to 1 GHz (not shown). We have also achieved upper sideband (USSB) modulation for a different experimental condition of *H*_app_ = 200 Oe and *φ* = 260°. At this condition, the STNO frequency shows a blue shift with dc bias current [see supplementary information]. However, the signals obtained in that condition were having large linewidth. Hence, we will concentrate on LSSB in this study.

### Macrospin simulations

**Figure 3:**
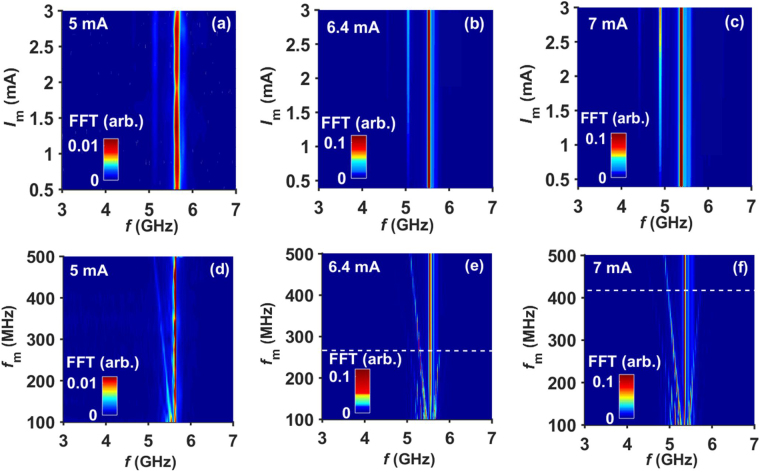
Macrospin simulation results: Map of power vs. frequency and modulation current for *f*_*m*_ = 500 MHz at (**a**) *I*_dc_ = 5 mA, (**b**) *I*_dc_ = 6.4 mA, and (**c**) *I*_dc_ = 7 mA. Map of power vs. frequency and modulation frequency for *I*_*m*_ = 1.2 mA at (**d**) *I*_dc_ = 5 mA, (**e**) *I*_dc_ = 6.4 mA, and (**f**) *I*_dc_ = 7 mA. The white dashed lines in (**e**) and (**f**), separate the NFAM and SSB regions.

The behavior of LSSB modulation has been reproduced by macrospin simulations performed at *T* = 300 K, as shown in Fig. 3. Similar to the experimental conditions, we have selected two bias currents from Fig. 1(e). These conditions are *I*_dc_ = 5 mA and 6.4 mA, corresponding to the sub-threshold region and above the threshold region, respectively. The simulated results show excellent qualitative agreement with the experimental results of Fig. 2. At *f*_*m*_ = 500 MHz, LSSB can be achieved for *I*_*m*_ = 0.5–3 mA in both operating regions. At *I*_*m*_ = 1.2 mA, LSSB can be achieved for entire range of *f*_*m*_ = 100–500 MHz for sub-threshold region of *I*_dc_ = 5 mA. For above threshold region of *I*_dc_ = 6.4 mA, LSSB can be achieved for *f*_*m*_ > 275 MHz at *I*_*m*_ = 1.2 mA [white dashed line in Fig. 3(e)]. In simulations, we also studied the modulation behavior at a much higher bias current of *I*_dc_ = 7 mA, and found that LSSB can be achieved at relatively high *f*_*m *_> 425 MHz [white dashed line in Fig. 3(f)] for *I*_*m*_ = 1.2 mA. In fact, the onset *f*_*m*_ where a clear SSB can be seen above the threshold current increases with *I*_dc_, in qualitative agreement with experiment. At a fixed modulation current, the onset of LSSB is also found to be related to the difference (*f*_*max*_ − *f*_0_) in simulations in agreement with experimental results [see supplementary information].

### Agreement with theory of nonlinear frequency and amplitude modulation

**Figure 4:**
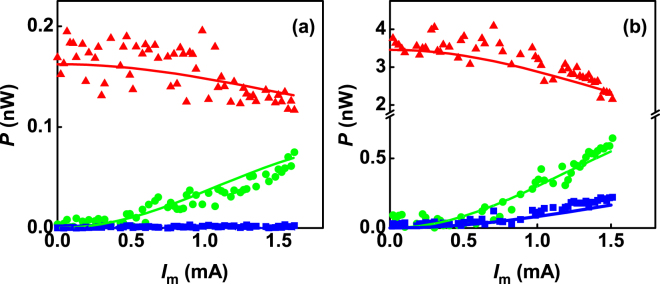
Integrated power of the carrier (red triangles) and of the first-order upper (blue squares) and lower (green circles) sidebands for *f*_*m*_ = 200 MHz at (**a**) *I*_dc_ = 4.4 mA and (**b**) 6.4 mA, respectively. The calculated integrated power as predicted by NFAM is shown by the solid lines.

The generation of LSSB with complete suppression of the upper sideband is very striking. We will show that this is a consequence of combined nonlinear frequency and amplitude modulation (NFAM)^33^. NFAM theory predicts an unequal amplitude of sidebands and has been successfully applied to nanocontact STNOs^18,19 as well as spin Hall nano-oscillators23^. In Fig. 4(a,b) we show the experimental behavior of the power of the carrier and of the first-order sidebands with modulation current measured at *f*_*m*_ = 200 MHz for *I*_dc_ = 4.4 mA and 6.4 mA, respectively. As can be seen, the upper sideband has significantly less power than the lower sideband.

We will now describe the behavior of the power of the carrier and sidebands with modulation current using NFAM theory. Assuming that the frequency is nonlinear up to fourth order and the amplitude is nonlinear up to third order, the NFAM spectrum can be written as^33^:

1 $$\begin{array}{rcl}S(f) & = & \frac{1}{4}\sum _{h=0}^{3}{\gamma }_{h}\sum _{n,m,p,q=-\infty }^{\infty }{J}_{n}({\beta }_{1}){J}_{m}({\beta }_{2}){J}_{p}({\beta }_{3}){J}_{q}({\beta }_{4})\\  &  & \times [\delta (f-{f}_{c}^{I}-(n+2m+3p+4q+k){f}_{m})\\  &  & +\delta (f-{f}_{c}^{I}-(n+2m+3p+4q-k){f}_{m})\\  &  & +\,\delta (f+{f}_{c}^{I}-(n+2m+3p+4q+k){f}_{m})\\  &  & +\delta (f+{f}_{c}^{I}-(n+2m+3p+4q-k){f}_{m})],\end{array}$$

where ${\beta }_{1}={k}_{1}{I}_{m}/{f}_{m}+3{k}_{3}{I}_{m}^{3}/4{f}_{m}$, ${\beta }_{2}={k}_{2}{I}_{m}^{2}/4{f}_{m}+{k}_{4}{I}_{m}^{4}/4{f}_{m}$, ${\beta }_{3}={k}_{3}{I}_{m}^{3}/12{f}_{m}$ and ${\beta }_{4}={k}_{4}{I}_{m}^{4}/32{f}_{m}$ are frequency modulation indices of different order, and ${\gamma }_{0}={\lambda }_{0}+{\lambda }_{2}{I}_{m}^{2}/2$, ${\gamma }_{1}={\lambda }_{1}{I}_{m}+3{\lambda }_{3}{I}_{m}^{3}/4$, ${\gamma }_{2}={\lambda }_{2}{I}_{m}^{2}/2$, and ${\gamma }_{3}={\lambda }_{3}{I}_{m}^{3}/4$ are amplitude modulation parameters. The frequency spectrum S(*f*) consists of a shifted carrier central frequency ${f}_{c}^{I}$ = *k*_0_ + *k*_2_ ${I}_{m}^{2}$ + 3 ${k}_{4}{I}_{m}^{4}/8$ + higher order terms. The infinite number of sidebands are considered to be symmetrically located at ${f}_{c}^{I}\pm l{f}_{m}$, where *l* = *n* + 2*m* + 3*p* + 4*q* ± h is a positive integer that represents the sideband order. The amplitude modulation (*k*_*i*_, where *i* =  1, 2, 3, 4) and frequency modulation (*λ*_*i*_, where *i* =  1, 2, 3) indices are calculated from the fourth and third-order polynomial fits to the free-running behavior of the frequency and amplitude with the bias current. These fits and the corresponding calculation of the amplitude and frequency modulation indices are shown in the supplementary information online.

We keep *I*_*m*_ within the 0–1.5 mA range so that the modulation acts as a small perturbation. As expected from modulation theory, the carrier power decreases and the lower and upper sideband power increases with the modulation current. The NFAM theory quantitatively reproduces the same dependence on the modulation current *I*_*m*_ [Fig. 4(a,b)] as *I*_*m*_ increases. However, for *I*_dc_ = 4.4 mA, the upper sideband is always completely suppressed and only the lower sideband is present. Such asymmetric sideband power is a consequence of the STNO’s nonlinearity. At *I*_dc_ = 6.4 mA, the LSSB power is nonzero at 200 MHz.

### Effect of field-like torque on SSB generation

**Figure 5:**
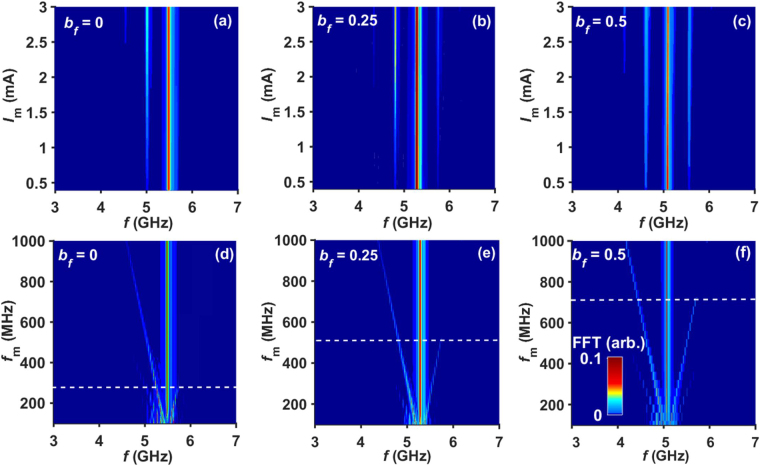
Macrospin simulation results: Map of power vs. frequency and modulation current at *I*_dc_ = 6.4 mA and *f*_*m*_ = 500 MHz for (**a**) *b*_*f*_ = 0, (**b**) *b*_*f*_ = 0.25, and (**c**) *b*_*f*_ = 0.5. Map of power vs. frequency and modulation frequency for *I*_*m*_ = 1.2 mA at (**d**) *b*_*f*_ = 0, (**e**) *b*_*f*_ = 0.25, and (**f**) *b*_*f*_ = 0.5. The white dashed lines in (**d**) and (**e**) separate the NFAM and SSB regions. The colorbar shown in (**f**) applies to all (**a**–**f**).

In order to further explore the origin of SSB in our experiment, we investigated the influence of field-like torque on SSB modulation using macrospin simulations. Figures 5(a–f) show the effect of the ratio of field-like torque to spin transfer torque, *b*_*f*_ = 0, 0.25, and 0.50 over the broad range of *f*_*m*_ = 150–1000 MHz and *I*_*m*_ = 0.5–3 mA. It is clear from the comparison that, at higher field-like torque, no clear SSB modulation is observed. At low values of *b*_*f*_ = 0.25, the threshold *f*_*m*_ increases drastically from 275 MHz to 525 MHz, as compared to the condition of *b*_*f*_ = 0. Utilizing a higher *b*_*f*_ = 0.5 increases the onset *f*_*m*_ for seeing the SSB to 725 MHz. This behavior of the LSSB with field-like torque is expected on the basis of the following arguments: LSSB is mainly caused by the large-amplitude nonlinearity and weak red-shift of frequency shown in Fig. 1. The field-like term affects the frequency tunability with the bias current [see supplementary Fig. 4(a)]^30,34,35^. Hence, for higher field-like terms, the frequency of the upper sideband can lie below *f*_max_ with finite power, leading to the disappearance of LSSB. Thus, the observation of LSSB in our experimental results indicates the presence of smaller field-like torque, which is consistent with our earlier work on similar devices^30,36^.

## Conclusion

We have here explored the nonlinear frequency and amplitude modulation behavior in order to obtain SSB modulation in an MTJ-based STNO. We demonstrated LSSB for both the sub-threshold and above-threshold regimes at modulation current in the range 0.3–1.5 mA. Successful LSSB was also achieved for modulation frequencies *f*_*m*_ ranging from 150 MHz to 1 GHz, indicating robust LSSB modulation in these systems. The performance of the STNOs as an LSSB generator can be tuned with operating conditions as well as with the field-like torque term present in MTJ-based devices. Given their high data rate, smaller size, and easier implementation, SSB modulation through MTJ-based STNOs may potentially meet the demand for integration on microchip-based communication devices to replace existing complex SSB generators.

## Methods

### Experimental procedure

The dc bias current was applied to the MTJ device using a bias tee. A resistive microwave power divider (0–12.4 GHz) was used between the bias tee and amplifier to send the modulating signal from an external signal source (a Rohde & Schwarz SMB 100 A signal generator). The modulation current was varied from 0 mA to 1.5 mA and the modulation frequency was varied in the 50 MHz–1 GHz range. The signal was amplified and detected with a spectrum analyzer. The amplifier had a gain of 52 dB and a working range of 1–18 GHz. All the data have been corrected to take into account the amplification, losses due to the power divider, losses in the transmission line, and losses due to reflection at the STNO (impedance mismatch). The reflection due to impedance mismatch is calculated from the scattering matrix element *S*_11_, measured with a vector network analyzer^15^. A projected field magnet mounted on a stepper motor was used to vary the in-plane field angle. The results were reproduced in a number of devices (>10).

### Macrospin Simulation

Macrospin simulations were performed by solving the Landau–Lifshitz–Gilbert–Slonczewski (LLGS) equation. The fourth-order Runge–Kutta method was used for temporal discretization of the LLGS equation, which was then integrated in small time steps *dt* to obtain an approximate numerical solution. The parameters used for the simulation are as follows: saturation magnetization *M*_sat_ = 10^6^ A/m; diameter of the device *D* = 240 nm; polarization efficiency *P* = 0.65; thickness of the free layer *t*_fl_ = 3.5 nm; and Gilbert damping parameter *α* = 0.022. The fixed-layer polarization was taken along the $\hat{x}$ direction. The device diameter was used as a fitting parameter to match the threshold current with experiments. The parameters used for the simulation are similar to those in our earlier work^36^. An interlayer exchange coupling (*H*_*IEC*_) of 117 Oe, and field angle of 188° was used in the simulations to match the experimental frequency of the STNO. All simulation were performed at *T* = 300 K. To include the effect of temperature, a time-varying random field was also introduced in the system, adding to the net effective field. This random noise was scaled numerically using the formulation given by William Fuller Brown^37^. During the simulation, the behavior of magnetization under different external perturbations was recorded in the time domain. This data was later converted to the frequency domain using a Fast Fourier transform (FFT), for comparison with the experimental results.

## Electronic supplementary material

Supplementary Information
