# Supplementary material for: A high-speed single sideband generator using a magnetic tunnel junction spin torque nano-oscillator
Source: Sci Rep. 2017 Oct 18;7:13422. doi: 10.1038/s41598-017-13551-5 (PMC5647387; doi:10.1038/s41598-017-13551-5)
Supplement: Supplementary file 1 — Supplementary Information [file 41598_2017_13551_MOESM1_ESM.pdf]

Supplementary material: A high-speed single sideband generator using a magnetic tunnel junction spin torque nano-oscillator

Raghav Sharma[1,*], **Naveen Sisodia**[1], **Ezio Iacocca**[2,3], **Ahmad A. Awad**[4], **Johan Åkerman**[4,5], **and P. K Muduli**[1,+]

[1]Department of Physics, Indian Institute of Technology, Hauz Khas, New Delhi-110016, India
[2]Department of Physics, Division for Theoretical Physics, Chalmers University of Technology, 412 96, Gothenburg, Sweden
[3]Department of Applied Mathematics, University of Colorado, Boulder, Colorado 80309, US.
[4]Department of Physics, University of Gothenburg, 41296, Gothenburg, Sweden
[5]Materials and Nanophysics, School of Engineering Sciences, KTH-Royal Institute of Technology, Electrum 229, 164 40 Kista, Sweden
[*]sharmaraghav66@yahoo.com
[+]muduli@physics.iitd.ac.in

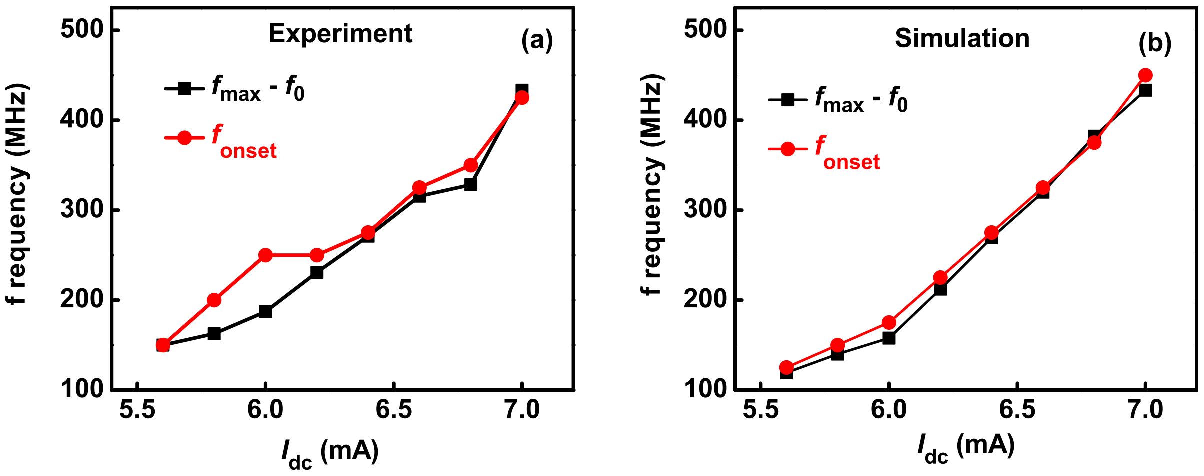

Figure 1. Comparison of onset modulation frequency, f_{onset} for observing lower single side band (LSSB) and (f_{max} - f_0) with varying dc bias current at $H_{\text{app}} = 450$ Oe, φ = 190° for (a) experiment and (b) macrospin simulations.

Onset of LSSB

The onset modulation frequency, f_{onset} for observing lower single side band (LSSB) generation in STNOs can be predicted from the frequency difference (f_{max} - f_0). Here, f_{max} is the resonance frequency at low bias current or the maximum operating frequency of the STNO and f_0 is the current dependent STNO frequency as defined in the main text. Figure 1 (a) shows the experimentally measured f_{onset} and calculated frequency difference (f_{max} - f_0), while Fig. 1 (b) shows corresponding results from macrospin simulations. The agreement shows that f_{onset} at any operating dc current can be obtained from the frequency difference, ($f_{max} - f_0$).

Upper single side band generation

We have achieved upper single sideband (USSB) modulation for an experimental condition of $H_{\text{app}} = 200$ Oe and $\varphi = 260°$. At this condition, the STNO frequency shows a blue shift with dc bias current. The free running STNO frequency and power at $H_{\text{app}} = 200$ Oe and $\varphi = 260°$ are shown in Fig. 2(a) and (b), respectively. The blue shift of STNO frequency with bias current we observe here is likely due to magnetization precession in synthetic antiferromagnetic (SyF) layer[1], as this behavior was not reproduced in our macrospin simulations of the free layer. Figure 2(c) is an example of USSB spectra at $I_{\text{dc}} = 4.4$ mA, $I_{\text{m}} = 1.2$ mA and $f_{\text{m}} = 700$ MHz. As expected from the NFAM formulation, USSB is produced in this case due to the blue shift of STNO frequency with bias current and strong amplitude non-linearity. However, the free-running linewidth at this bias condition is relatively high, which makes the modulation experiment difficult due to the overlap of the carrier and sidebands.

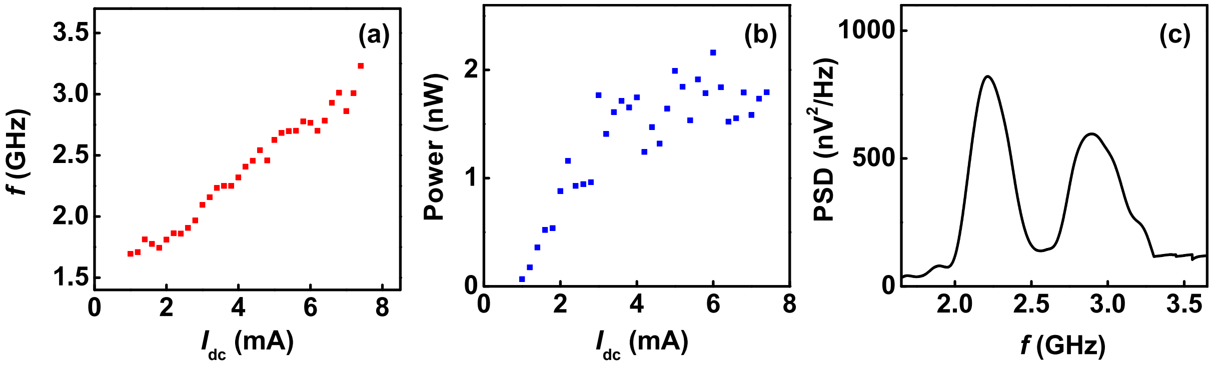

Figure 2. (a) Frequency and (b) Power vs d.c. current (in the absence of any RF current) measured at $H_{\text{app}} = 200$ Oe, φ = 260°. (c) Sample spectrum showing upper single sideband modulation at $I_{\text{dc}} = 4.4$ mA at $I_{\text{m}} = 1.2$ mA and $f_{\text{m}} = 700$ MHz

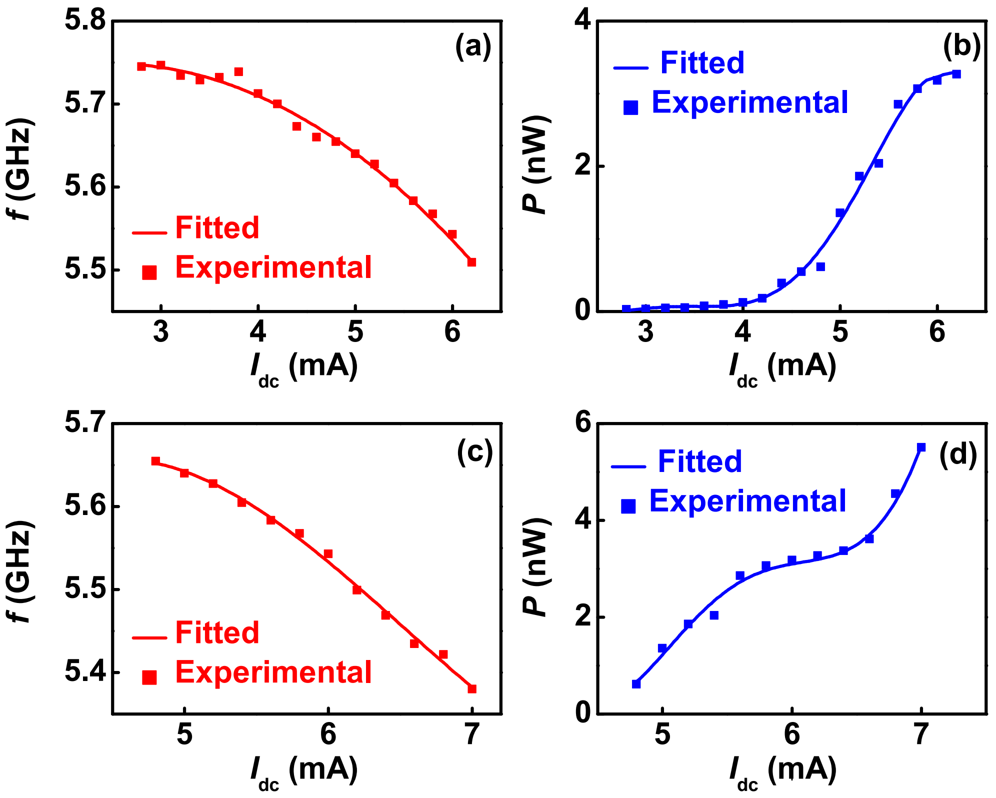

Figure 3. Dependence of (a) the oscillator frequency and (b) power with dc bias current around $I_{\mathrm{dc}} = 4.4$ mA. Similarly, (c) and (d) show the dependence of oscillator frequency and power, respectively, with dc bias current around $I_{\mathrm{dc}} = 6.4$ mA. The solid lines are polynomial fits to the experimental data.

Determination of frequency and amplitude modulation sensitivity coefficients from free-running properties

In the NFAM formulation,[2,3] the instantaneous frequency is assumed to depend nonlinearly on the modulating signal:

$$f_i(t) = k_0 + k_1 m(t) + k_2 m(t)^2 + k_3 m(t)^3 + k_4 m(t)^4 + \ldots \tag{1}$$

where $m(t)$ is the modulating signal and the coefficients k_i represent the i-th order frequency modulation sensitivity. Similarly, the output amplitude A_c is given by:

$$A_c(t) = \lambda_0 + \lambda_1 m(t) + \lambda_2 m(t)^2 + \lambda_3 m(t)^3 + \ldots \tag{2}$$

where λ_i is the i-th order amplitude sensitivity coefficient. The coefficients k_i and λ_i describe the nonlinear current dependence of f_i and A_c of the free-running STNO. We use sine wave modulation, $m(t) = I_m sin(2\pi f_m t)$, where I_m is the amplitude and f_m is the frequency of modulation.

Table 1. Frequency modulation sensitivity coefficients from the polynomial fits of the frequency of the free-running STO.

I_{dc} (mA)	k_0 (GHz)	k_1 (GHz/mA)	k_2 (GHz/mA^2)	k_3 (GHz/mA^3)	k_4 (GHz/mA^4)
4.4	5.653±0.003	-0.134±0.004	0.0331±0.0018	-0.011±0.001	-0.0009±0.0003
6.4	5.469±0.003	-0.184±0.011	-0.0233±0.0018	0.011±0.002	-0.0014±0.0002

Figure 3 shows the fitting of the experimental free-running STNO frequency and power to Eq. (1) and the square of Eq. (2) up to the fourth and third-order nonlinear polynomial, respectively, around $I_{\mathrm{dc}} = 4.4$ mA and 6.4 mA. The range of dc current taken around the bias point $I_{\mathrm{dc}} = 4.4$ mA and 6.4 mA is same as the range of modulation current applied in the experiment *i.e.*, ±1.6 mA. For the case of $I_{\mathrm{dc}} = 6.4$ mA, the upper limit of fitting is restricted by the maximum applied dc current, namely $I_{\mathrm{dc}} = 7$ mA in the modulation experiment, for safer operation of the STNO device. The values of frequency and amplitude modulation sensitivity coefficients derived from Fig. 3 are shown in Table 1 and 2, respectively.

Table 2. Amplitude modulation sensitivity coefficients from the polynomial fits of the amplitude of the free-running STO.

I_{dc} (mA)	λ_0 (pW$^{1/2}$)	λ_1 (pW$^{1/2}$/mA)	λ_2 (pW$^{1/2}$/mA^2)	λ_3 (pW$^{1/2}$/mA^3)
4.4	13.03±0.37	5.61±0.23	1.32±0.08	0.29±0.05
6.4	59.24±0.32	9.04±0.54	5.64±0.17	1.68±0.36

Macrospin simulation of STNO frequency vs. bias current with varying field-like torque

Macrospin simulations were performed by solving the Landau–Lifshitz–Gilbert–Slonczewski (LLGS) equation[4,5]:

$$\frac{d\hat{m}}{dt} = -\gamma(\hat{m}\times\vec{H}_{\mathrm{eff}}) + \alpha(\hat{m}\times\frac{d\hat{m}}{dt}) - \gamma\frac{J\hbar P}{2eM_s t_{\mathrm{fl}}(1+P^2\cos\phi)}\left[\hat{m}\times(\hat{m}\times\hat{e}_p) + b_f(\hat{m}\times\hat{e}_p)\right] \tag{3}$$

Here, $\hat{m}$ is the normalized magnetic moment, γ is the gyromagnetic ratio of the electron, J is the spin polarized current density, e is the electronic charge and ϕ is the angle between the free and fixed layers. The saturation magnetization of the free layer M_{s} and the Gilbert damping constant α were taken to be 10^6 A/m and 0.022, respectively. The thickness of the free layer, $t_{\mathrm{fl}} = 3.5$ nm. The magnetization of the fixed layer, $\hat{e}_p$, is taken along $\hat{x}$ axis with a polarization efficiency, $P = 0.65$. The value of the field-like torque is changed by varying the ratio between the field like torque and the spin transfer torque, b_f to check the tunability of the free-running STNO frequency (f) vs. dc bias current (I_{dc}). $\vec{H}_{\mathrm{eff}}$ is the effective field acting on the STNO, which includes a contribution from the applied external field ($\vec{H}_{\mathrm{ext}}$), the demagnetizing field ($\vec{H}_{\mathrm{demag}}$), interlayer exchange coupling ($\vec{H}_{\mathrm{IEC}}$) and the thermal field ($\vec{H}_{\mathrm{therm}}$). $\vec{H}_{\mathrm{app}}$ is applied in the plane of the sample having only $\hat{x}$ and $\hat{y}$ components. $\vec{H}_{\mathrm{demag}}$ is defined as:

$$\vec{H}_{\mathrm{demag}} = -M_{\mathrm{s}}(N_{\mathrm{x}}m_{\mathrm{x}}\hat{x} + N_{\mathrm{y}}m_{\mathrm{y}}\hat{y} + N_{\mathrm{z}}m_{\mathrm{z}}\hat{z}), \tag{4}$$

where, N_{x}, N_{y} and N_{z} are the demagnetization factors used to characterize the film geometry. The values of demagnetization factors have been approximated for the case of a thin circular disk where the thickness is much lower than the diameter[6]. The calculated values of N_x, N_y and N_z are 0.01125, 0.01125 and 0.9775, respectively.

An interlayer exchange coupling ($\vec{H}_{\mathrm{IEC}}$) of $\sim$ 117 Oe is included in the net effective field. The thermal field, $\vec{H}_{\mathrm{therm}}$ is defined according to Brown's approximation[7,8] by adding a random fluctuating field whose components in different directions satisfy the following criteria:

$$\langle H^i_{\mathrm{therm}}(t)H^j_{\mathrm{therm}}(t)\rangle = \frac{2k_{\mathrm{B}}T\alpha}{\gamma V\mu_0 M_s}\delta_{ij}\delta(t-t'), \tag{5}$$

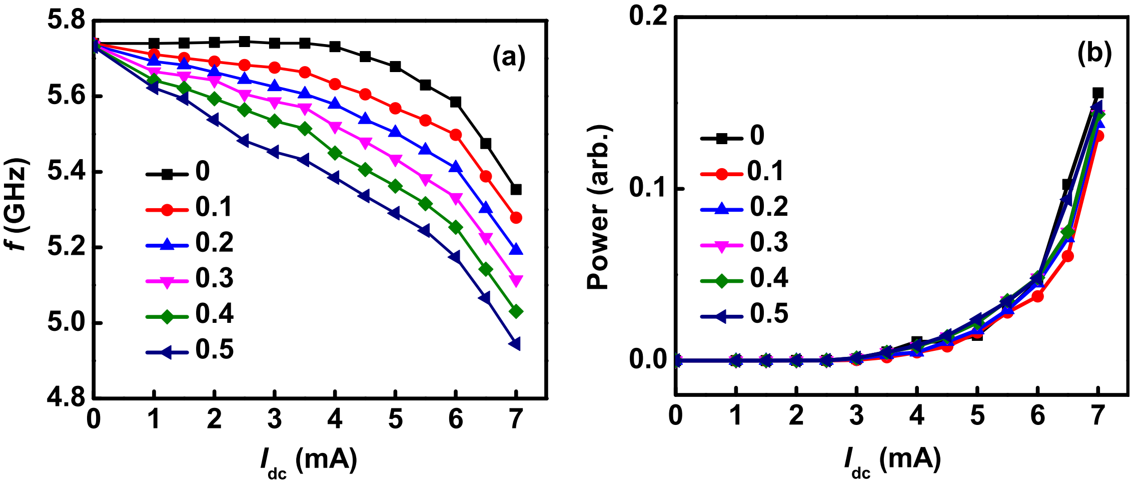

Figure 4. (a) STNO frequency and (b) Power vs. bias current for various values of the field-like torque to spin transfer torque ratio (b_{f}).

Using such a criteria leads to a Boltzmann distribution of energies at equilibrium condition. Here, the variables i and j refer to components of the time-varying thermal field in different directions. k_B is the Boltzmann constant, T is the temperature, V is the volume of the free layer and μ_0 the magnetic permeability of free space.

Figure 4(a) shows the f vs. I_{dc} data for varying b_f in the range of 0 to 0.5. For the case of $b_f = 0$, we found weak tunability of the STNO frequency with the bias current. However, as b_f increases, the current tunability becomes stronger. We did not see strong variation in the power with the increase in field-like torque [Fig. 4(b)], which is expected, as the field-like torque affects only the resonance frequency of the STNO[9–11]. The weak tunability for low values of b_f is the reason for observing LSSB in Fig. 5 of the main paper.

References

1. Monteblanco, E., Garcia-Sanchez, F., Gusakova, D., Buda-Prejbeanu, L. & Ebels, U. Spin transfer torque nano-oscillators based on synthetic ferrimagnets: Influence of the exchange bias field and interlayer exchange coupling. *J. Appl. Phys.* **121**, 013903 (2017).
2. Consolo, G. *et al.* A Generalized Model of Nonlinear Dynamics in Combined Frequency-Amplitude Modulators. *IEEE Trans. Magn.* **46**, 3629–3634 (2010).
3. Muduli, P. K. *et al.* Nonlinear frequency and amplitude modulation of a nanocontact-based spin-torque oscillator. *Phys. Rev. B* **81**, 140408 (2010).
4. Zhu, J. *et al.* Voltage-induced ferromagnetic resonance in magnetic tunnel junctions. *Phys. Rev. Lett.* **108**, 197203 (2012).
5. Zeng, T., Zhou, Y., Lin, K. W., Lai, P. T. & Pong, P. W. T. Spin-torque diode-based radio-frequency detector by utilizing tilted fixed-layer magnetization and in-plane free-layer magnetization. *IEEE Trans. Magn.* **51**, 1401204 (2015).
6. Osborn, J. A. Demagnetizing factors of the general ellipsoid. *Phys. Rev.* **67**, 351–357 (1945).
7. Brown Jr, W. F. Thermal fluctuations of a single-domain particle. *J. Appl. Phys.* **34**, 1319–1320 (1963).
8. Xiao, J., Zangwill, A. & Stiles, M. D. Macrospin models of spin transfer dynamics. *Phys. Rev. B* **72**, 014446 (2005).
9. Muduli, P. K., Heinonen, O. G. & Åkerman, J. Bias dependence of perpendicular spin torque and of free- and fixed-layer eigenmodes in MgO-based nanopillars. *Phys. Rev. B* **83**, 184410 (2011).
10. Heinonen, O. G., Stokes, S. W. & Yi, J. Y. Perpendicular Spin Torque in Magnetic Tunnel Junctions. *Phys. Rev. Lett.* **105**, 066602 (2010).
11. Petit, S. *et al.* Spin-Torque Influence on the High-Frequency Magnetization Fluctuations in Magnetic Tunnel Junctions. *Phys. Rev. Lett.* **98**, 077203 (2007).
